# Validation of Quantitative Ultrasound and Texture Derivative Analyses-Based Model for Upfront Prediction of Neoadjuvant Chemotherapy Response in Breast Cancer

**DOI:** 10.3390/jimaging11040109

**Published:** 2025-04-03

**Authors:** Adrian Wai Chan, Lakshmanan Sannachi, Daniel Moore-Palhares, Archya Dasgupta, Sonal Gandhi, Rossanna Pezo, Andrea Eisen, Ellen Warner, Frances C. Wright, Nicole Look Hong, Ali Sadeghi-Naini, Mia Skarpathiotakis, Belinda Curpen, Carrie Betel, Michael C. Kolios, Maureen Trudeau, Gregory J. Czarnota

**Affiliations:** 1 Department of Radiation Oncology, Sunnybrook Health Sciences Centre, Toronto, ON M4N 3M5, Canadadaniel.palhares@sunnybrook.ca (D.M.-P.); archya1010@gmail.com (A.D.); ali.sadeghi@sri.utoronto.ca (A.S.-N.); 2 Department of Radiation Oncology, University of Toronto, Toronto, ON M5T 1P5, Canada; 3 Physical Sciences, Sunnybrook Research Institute, Toronto, ON M4N 3M5, Canada; lakshmanan.sannachi@sunnybrook.ca; 4 Division of Medical Oncology, Department of Medicine, Sunnybrook Health Sciences Centre, Toronto, ON M4N 3M5, Canada; sonal.gandhi@sunnybrook.ca (S.G.); rossanna.pezo@sunnybrook.ca (R.P.); andrea.eisen@sunnybrook.ca (A.E.); ellen.warner@sunnybrook.ca (E.W.); maureen.trudeau@sunnybrook.ca (M.T.); 5 Department of Medicine, University of Toronto, Toronto, ON M5S 3H2, Canada; 6 Division of General Surgery, Department of Surgery, Sunnybrook Health Sciences Centre, Toronto, ON M4N 3M5, Canada; frances.wright@sunnybrook.ca (F.C.W.); nicole.lookhong@sunnybrook.ca (N.L.H.); 7 Department of Surgery, University of Toronto, Toronto, ON M5T 1P5, Canada; 8 Department of Electrical Engineering and Computer Sciences, Lassonde School of Engineering, York University, Toronto, ON M3J 1P3, Canada; 9 Department of Medical Imaging, Sunnybrook Health Sciences Centre, Toronto, ON M4N 3M5, Canada; mia.skarpathiotakis@sunnybrook.ca (M.S.); belinda.curpen@sunnybrook.ca (B.C.);; 10 Department of Medical Imaging, University of Toronto, Toronto, ON M5T 1W7, Canada; 11 Department of Physics, Ryerson University, Toronto, ON M5B 2K3, Canada; mkolios@torontomu.ca; 12 Department of Medical Biophysics, University of Toronto, Toronto, ON M4N 3M5, Canada

**Keywords:** radiomics, machine learning, quantitative ultrasound

## Abstract

This work was conducted in order to validate a pre-treatment quantitative ultrasound (QUS) and texture derivative analyses-based prediction model proposed in our previous study to identify responders and non-responders to neoadjuvant chemotherapy in patients with breast cancer. The validation cohort consisted of 56 breast cancer patients diagnosed between the years 2018 and 2021. Among all patients, 53 were treated with neoadjuvant chemotherapy and three had unplanned changes in their chemotherapy cycles. Radio Frequency (RF) data were collected volumetrically prior to the start of chemotherapy. In addition to tumour region (core), a 5 mm tumour-margin was also chosen for parameters estimation. The prediction model, which was developed previously based on quantitative ultrasound, texture derivative, and tumour molecular subtypes, was used to identify responders and non-responders. The actual response, which was determined by clinical and pathological assessment after lumpectomy or mastectomy, was then compared to the predicted response. The sensitivity, specificity, positive predictive value, negative predictive value, and F1 score for determining chemotherapy response of all patients in the validation cohort were 94%, 67%, 96%, 57%, and 95%, respectively. Removing patients who had unplanned changes in their chemotherapy resulted in a sensitivity, specificity, positive predictive value, negative predictive value, and F1 score of all patients in the validation cohort of 94%, 100%, 100%, 50%, and 97%, respectively. Explanations for the misclassified cases included unplanned modifications made to the type of chemotherapy during treatment, inherent limitations of the predictive model, presence of DCIS in tumour structure, and an ill-defined tumour border in a minority of cases. Validation of a model was conducted in an independent cohort of patient for the first time to predict the tumour response to neoadjuvant chemotherapy using quantitative ultrasound, texture derivate, and molecular features in patients with breast cancer. Further research is needed to improve the positive predictive value and evaluate whether the treatment outcome can be improved in predicted non-responders by switching to other treatment options.

## 1. Introduction

Neoadjuvant chemotherapy is commonly used in locally advanced breast cancer and selected patients with human epidermal growth factor receptor 2 (HER2) overexpressed and triple negative breast cancer [1]. The response rate to neoadjuvant chemotherapy ranges from 60 to 90% [2]. Patients who had tumour progression during neoadjuvant chemotherapy may benefit from either neoadjuvant radiotherapy or proceeding directly to surgery if the tumour is still operable [3]. Addition of a targeted therapy such as pertuzumab/trastuzumab or immunotherapy such as pembrolizumab to chemotherapy can also increase the response rate in HER2 overexpressed and triple negative breast cancer, respectively [4,5]. In light of the availability of these alternate management strategies, early identification of patients with predicted poor response to chemotherapy may improve outcome by allowing an early change in treatment.

Ultrasound has been used for several years for both diagnostic and therapeutic purposes. It is highly sensitive to the variations in micro-structural properties of tissues at various size scales. Several studies have utilized texture features of ultrasound B-mode image for tissue characterization application [6,7,8]. In addition to the B-mode image, quantitative ultrasound parameters extracted from RF data have been used for tissue characterization, breast lesion assessment, and monitoring tumour response early and during the course of treatment [9,10,11,12,13,14,15,16,17]. In RF-mode imaging, tissue properties are interpreted from variations in the RF-spectrum, whereas standard B-mode images are derived from the envelope of RF signals, utilizing only a fraction of the information available in the RF signal. QUS methods provide more detailed information about tissue properties at a cellular level through spectral analysis of the RF signal based on fundamental acoustic attributes of ultrasound backscatter.

Several studies have investigated the utilization of the envelop statistics of raw RF data including Rayleigh Nakagami, K distribution, Kolmogorov–Smirnov statistics, and the symmetrical Kullback–Leibler in tissue characterization applications. These studies have reported an AUC of 0.92 [9] for breast lesion characterization and AUC values of 0.84 and 0.9 for detecting breast cancer response to NAC after the first and third doses of drugs, respectively [13]. Several studies have investigated deep learning approaches for tissue characterization and treatment response prediction using raw RF ultrasound data or conventional ultrasound images. These studies reported AUC values ranging from 0.81 to 0.93 for predicting breast cancer treatment response during treatment, despite the limited cohort size [18,19,20,21]. Recently, texture feature from ultrasound backscatter parametric images has been demonstrated for the early detection of breast cancer treatment response, with an AUC of 0.81–0.94 [17].

In our previous study, several QUS parameters including mid-band fit, (MBF), spectral slope (SS), 0 MHz intercept, average scatterer diameter (ASD), and average acoustic concentration (AAC) extracted from tumour ultrasound RF data were investigated for evaluating tumour response to treatment early after starting NAC. These parameters showed a significant correlation with tumour response [22]. QUS parameters are related to several tissue properties such as scatterer size, scatterer orientation, scatterer concentration, and their elastic properties [23,24]. Combining QUS parameters with grey-level co-occurrence matrix (GLCM)-based texture parameters extracted from QUS parametric maps improved tumour response prediction with an accuracy of 78%, 90%, and 92% at weeks 1, 4, and 8 after the start of treatment, respectively [22].

Recently, models combining quantitative ultrasound, GLCM-based texture and texture derivative techniques were investigated to predict the tumour response to neoadjuvant chemotherapy before starting treatment in 100 breast cancer patients [25]. Based on these parameters, three machine learning algorithms were developed using linear discriminant, k-nearest-neighbours, and support vector machine. The best performance was obtained using k-nearest neighbours, with sensitivity, specificity, and accuracy of predicting treatment response at 87%, 81%, and 82%, respectively.

Several studies have demonstrated significant correlation between breast cancer molecular features and their pathological response to chemotherapy treatment [4,26,27,28]. In our most recent study, we investigated the performance of the pre-treatment breast tumour response prediction by combining molecular features with quantitative ultrasound, tumour core-margin, texture, and texture derivative analysis techniques in 208 breast cancer patients [29]. Two standard classification algorithms using KNN and a support vector machines-radial basis function (SVM-RBF) were evaluated. The best classification performance was obtained using an SVM-RBF classifier with a combination of QUS texture derivative from a tumour core and margin and molecular parameters with a sensitivity, specificity, and accuracy of 79%, 86%, and 85%, respectively. The aim of this study was to validate that previously developed QUS-Texture-derivate analyses based SVM-RBF model in a separate cohort of patients who received neoadjuvant chemotherapy for breast cancer. Such validation is needed to evaluate the performance of a predictive model [29] with regard to a defined target population or clinical setting, before it can be used in clinical practice [30].

## 2. Material and Methods

### 2.1. Developmental Cohort

Development of the QUS and texture derivate-based prediction model was performed on 100 patients with locally advanced breast cancer patients who received neoadjuvant chemotherapy [25]. Locally advanced breast cancer patient data were included from patients with histologically proven adenocarcinoma of the breast, clinical stage T3-T4 or N2-3 (AJCC 8th edition) [31]. Response assessment was carried out on the basis of the clinical/pathological tumour response determined at the end of their neoadjuvant treatment and surgery if it was performed [22]. Response classification included both complete or partial response and was defined as the disappearance of all target lesions or at least a 30% decrease in the diameter of the target lesions (RECIST criteria), based on pre-treatment measurement via magnetic resonance imaging of breast and the pathological examination following surgery [32]. Non-response was defined as less than a 30% decrease in tumour size and incorporated both stable disease and progressive disease. If no malignant cells were identified from the site of tumour after surgery, this would be classified as complete response. The QUS, texture, and texture derivative parameters were estimated from the tumour core region from the ultrasound RF data acquired prior to the treatment. Three classification algorithms using standard classifiers such as Fisher’s linear discriminant, k- nearest neighbours, and a radial-basis-function support vector machine were investigated. The best classification performance was obtained with the k-nearest neighbour classifier, with a sensitivity, specificity, and accuracy of 87%, 81%, and 82%, respectively.

**Figure 1:**
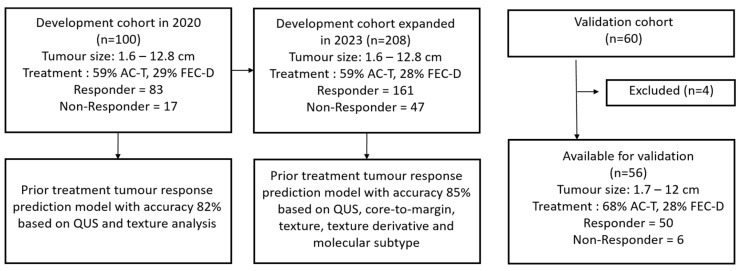
Flow charts of the developmental and validation cohort. AC-T = doxorubicin and cyclophosphamide, followed by paclitaxel. FEC-D = 5-Fluouracil, epirubicin, and cyclophosphamide, followed by docetaxel.

In order to improve the accuracy of the prediction model, the developmental cohort was expanded to 208 patients and additional features such as texture and texture-derived features from the tumour core and 5 mm margin, and a tumour molecular subtype were utilized [29]. In the updated developmental cohort, 59% of patients received doxorubicin, cyclophosphamide followed by paclitaxel, while 28% of patients received 5-fluouracil, epirubicin, and cyclophosphamide followed by docetaxel. Thirty-one percent of patients received trastuzumab in the neoadjuvant setting (Supplementary Table S3). A total of 10 optimal features including QUS texture derivative parameters from the tumour core and margin regions, and tumour molecular subtypes were used in the model, which was designed to predict the response after the entire course of neoadjuvant chemotherapy (Supplementary Table S2). Figure 1 shows flow charts of the developmental and validation cohort.

This study was performed in accordance with appropriate guidelines of the Sunnybrook Research Ethics Board at Sunnybrook Health Sciences Centre (SHSC), Toronto, Canada. All experimental protocols were reviewed and approved by the Sunnybrook Research Institute Research Ethics Board before commencing the study. Informed consent for participation was obtained from all subjects involved in the study.

### 2.2. Ultrasound Data Collection

Quantitative ultrasound data collection was performed by sonographers experienced in breast imaging using a Sonix RP clinical system (Ultrasonix, Vancouver, BC, Canada) with an L14-5/60 linear transducer (central frequency 6.5 MHz, bandwidth range 3.0–8.5 MHz). The sonographer defined the tumour region of interest for QUS, texture, and texture derivative estimation from the tumour core and 5 mm tumour-margin regions. In our previous study, tumour-margin analysis of QUS parametric images acquired from locally advanced breast cancer patients was investigated for the prediction of tumour response to neoadjuvant chemotherapy before the start of treatment, based on the hypothesis that the margin may account for the presence of microscopic infiltration from the primary tumour into the surrounding normal tissue [33]. The result demonstrated that, in addition to tumour core, QUS analysis of a 5 mm tumour surrounding region improved tumour response prediction performance.

This delineation of region of interest was verified by an expert breast radiologist and the principal investigator. Tumour size was defined as the maximal dimension of the tumour by ultrasound. Any disagreement was resolved by discussion and consensus. Tumours were volumetrically scanned and frames nominally 0.5 cm apart from each other were selected for analysis. If the whole tumour was too large to be included entirely as the region of interest, we selected a region most representative of the entire tumour by imaging an area where the signal was similar to the majority of the tumour. For larger tumours, wherever possible, we altered the imaging magnification or repositioned the transducer to include the shorter tumour axis in cross section, thereby covering the longer axis through volumetric data acquisition.

### 2.3. Quantitative Ultrasound Parameters

**Figure 2:**
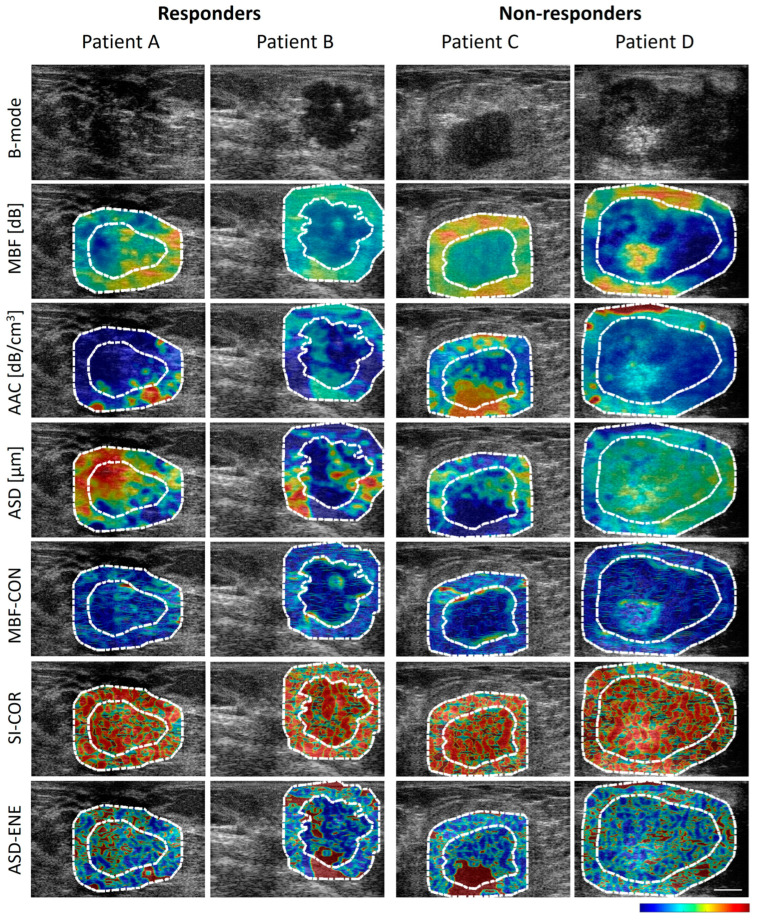
Quantitative ultrasound and texture images of response group. Representative B-mode and parametric images with the tumour core region (region within inner dotted white line) and margin region (region between inner and outer dotted white lines), the regions of responder (Patient A and B) and non-responder (Patient C and D). The white scale bar in ultrasound image represents 5 mm. The colour bar represents the scale for the MBF parameter of −9.6 to 35.9 dB, for the AAC parameter of 20.4 to 179.6 dB/cm^3^, for the ASD parameter of 85 to 198 µm, for the MBF-CON texture parameter of 0 to 1, for the SI-COR texture parameter of −0.48 to 1, and for the ASD-ENE texture parameter of 0 to 1. MBF: mid-band fit; AAC: average acoustic concentration; ASD: average scatterer diameter; CON: contrast; SI: spectral intercept; COR: correlation; ENE: energy.

Details of the quantitative ultrasound, texture, and texture derivative parameter estimation were explained in previous work [29]. The spectral parameters including the mid-band fit (MBF), spectral slope (SS), and spectral intercept (SI) were calculated using linear regression analysis of normalized backscatter power spectrum obtained from tumour RF data. The average scatterer diameter (ASD) and average acoustic concentration (AAC) were derived from the backscatter coefficient by comparing measure data with a theoretically derived backscatter coefficient. Finally, the quantitative ultrasound parametric maps, including MBF, SS, SI, ASD, and AAC were constructed from the tumour core and 5 mm tumour surrounding regions (margin) using a sliding window analysis technique. The mean values of QUS parameters were calculated from both tumour core and margin regions. Two core-to-margin related parameters including core-to-margin ration and core-to-margin contrast ratio were calculated from the tumour core and 5 mm margin regions in QUS parametric maps. Four texture features including contrast, correlation, homogeneity, and energy were extracted from QUS maps using a grey-level co-occurrence matrix (GLCM)-based texture analysis method. Finally, four texture maps were created from each QUS parametric images by the GLCM method using a sliding window analysis. A second pass GLCM-based texture analysis was performed on texture maps, resulting in texture derivative features. A total of 201 features (attenuation, mean QUS, texture, core-to-margin, and texture derivative parameters) were extracted from tumour ultrasound RF data (Supplementary Figure S1 and Table S1). Representative ultrasound B-mode, QUS, and QUS texture images from both tumour core and 5 mm margin regions corresponding to responding and non-responding patients, acquired prior to neoadjuvant chemotherapy, are shown in Figure 2.

### 2.4. Statistical Analysis

The sensitivity, specificity, positive predictive value (PPV) and negative predictive value (NPV), and F1 score were calculated in the validation cohort by comparing the predicted response to the clinical/pathological response. Sensitivity was defined as the proportion of responders who were correctly predicted to be responders. Specificity was the proportion of non-responders who were correctly predicted to be non-responders. PPV was calculated as the proportion of patients who were responders, among patients who were predicted to be responders. NPV was defined as the proportion of patients who were non-responders after neoadjuvant chemotherapy, among patients who were predicted to be non-responders. F1 score, which is the harmonic mean of precision and recall, is used to evaluate the performance of the classification model particularly when the data set are imbalanced. Statistical analysis was conducted with IBM SPSS version 22 (IBM Corporation, Armonk, NY, USA).

## 3. Results

### 3.1. Patient and Tumour Characteristics

Sixty patients with locally advanced breast cancer were recruited in this validation study. Four patients were excluded for incomplete QUS data, leaving 56 for analysis. The median age of the 56 patients was 50 years old. The median initial primary tumour size was 3.7 cm. Tumours were grouped into four molecular subtype as in the previous study [29], including ERBB2+ (ER-, PR-, HER2+), triple negative (ER-, PR-, HER2-), Luminal-A (ER+ and/or PR+, HER2-), and Luminal-B (ER+ and/or PR+, HER2+). Among all patients, 10.7%, 23.2%, 44.6%, and 21.5% were ERBB2+, triple negative, Luminal-A, and Luminal-B, respectively. Among all the patients, 62.5% received doxorubicin–cyclophosphamide followed by paclitaxel with or without trastuzumab as the neoadjuvant chemotherapy. All patients who had HER-2 positive breast cancer received trastuzumab as part of their neoadjuvant treatment. None of the patients received immunotherapy. Details of the patient cohort including their age, ER status, progesterone receptor status, HER-2 receptor status, histology, tumour grade, pre-chemotherapy tumour size, and chemotherapy or target therapy regimen are described in Table 1.

### 3.2. Prediction of Response

Amongst the 56 patients, 50 (89.3%) had achieved a response after neoadjuvant chemotherapy. Among them, 47 patients were predicted to be responders using the model being tested. Among the 6/56 (10.7%) patients who did not achieve a response after neoadjuvant chemotherapy, four patients were predicted to be non-responders. The actual and predicted response of each patient is given in Table 2.

The sensitivity, specificity, PPV, NPV, and F1 score of predicting treatment response of all patients in the validation cohort were 94%, 67%, 96%, 57%, and 95%, respectively. A matrix describing the treatment prediction result is given in Supplementary Figure S2a.

A small number of patients had unplanned changes in their chemotherapy during treatment (changes in type chemotherapy regimen after prediction). Removing these patients from the data set, the sensitivity, specificity, PPV, NPV, and F1 score of predicting the treatment response of all patients in the validation cohort were 94%, 100%, 100%, 50%, and 97%, respectively. A matrix describing the treatment prediction result for this is also given in Supplementary Figure S2b.

### 3.3. Incorrect Prediction

**Figure 3:**
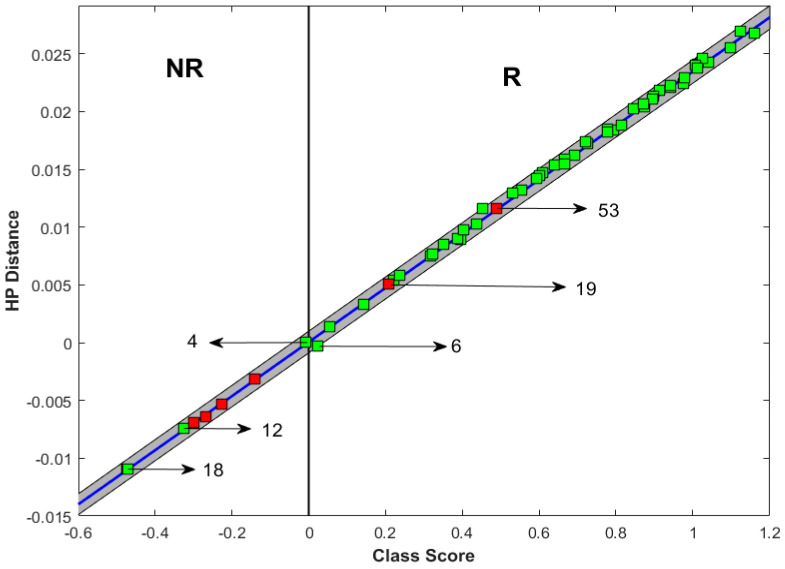
Treatment response prediction. The right side of the graph represents predicted responders (R zone), while the left side represents predicted non-responders (NR zone). Green and red squares were actual responders and non-responders, respectively. Therefore, misclassified patients were the red squares on the “R” zone and green squares in the “NR” zone. Here, the horizontal axis, the class score, represents the probability of predicting a particular patient’s treatment response based on the SVM-RBF model proposed in our previous study. The sign is assigned based on the response class prediction (positive for responding prediction and negative for non-responding prediction). The vertical axis, HP distance, represents the distance of new patient’s feature point from the QUS-radiomics support vector machine model hyperplane.

Due to the low positive predictive value for non-response, a detailed review of all of the patients who were misclassified as a non-responder was carried out. The misclassified patients and the class score of each patient are described in Figure 3. In this Figure 3, the horizontal axis, the class score represents the probability of predicting a particular patient’s treatment response based on the model with QUS and texture derivative parameters using the SVM-RBF classifier proposed in our previous study. The sign is assigned based on the response class prediction (positive for responding prediction and negative for non-responding prediction). The vertical axis, HP distance, represents the distance of a new patient’s feature point from the QUS-radiomics support vector machine model hyperplane.

A higher absolute value of the class score indicates a higher confidence of prediction by the model.

**Figure 4:**
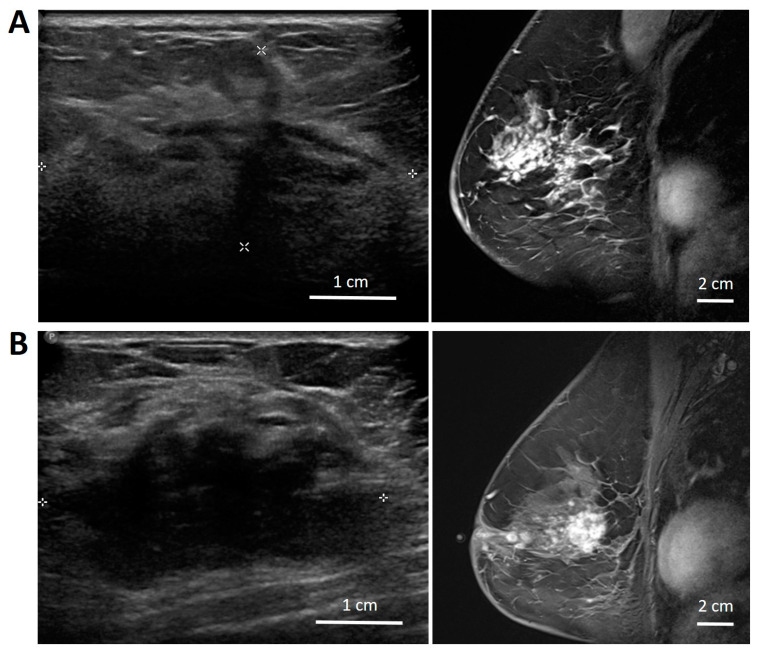
Example of an ultrasound (left) and MRI (right) images of patient 12 (**A**) and patient 19 (**B**) showing a tumour with heterogeneous, distorted, and poorly defined boundary structures.

Regarding the first patient (patient number 4 in Figure 3), the class score of prediction was close to 0, which means that the radiomics features were borderline (indeterminate) and did not strongly favour either responders or non-responders. In the second patient (patient number 12 in Figure 3), the tumour had a poorly defined border in ultrasound (Figure 4) and MRI, and it was possible that the delineated region of interest may not have accurately included the tumour plus a 5 mm margin. This could have influenced the accuracy of prediction. In the third patient (patient number 18 in Figure 3), the tumour was very close to the Cooper’s ligament. The region of interest was the tumour with a 5 mm margin, which in this case included the Cooper’s ligament. This might have affected the prediction accuracy, as the imaging parameters of the Cooper’s ligament can be very different from the usual 5 mm margin of adjacent breast tissue.

Other patients which were misclassified had high DCIS content in their tumours. Previous work has demonstrated that DCIS can be a confounder of QUS-based predictions and monitoring of response.

## 4. Discussion

To our knowledge, this is the first validation study of a model based on the combination of pre-treatment QUS, texture derivative, and molecular parameters to predict the response to neoadjuvant chemotherapy in an independent data set of early-stage or locally advanced breast cancer patients. The sensitivity, specificity, PPV, and NPV were 94%, 67%, 96%, and 57%, respectively. Removing patients who had unplanned changes in their chemotherapy resulted in a sensitivity, specificity, positive predictive value (PPV), and negative predictive value (NPV) of all patients in the validation cohort of 94%,100%, 100%, and 50%, respectively.

Previously published prediction models using positron emission tomography–computed tomography (PET-CT) radiomics features or CT radiomics features to predict response to neoadjuvant chemotherapy in breast cancer had sensitivity and specificity of 81.8% and 78.9% (PET-CT) and 59% and 84% (CT), respectively [34,35]. As discussed previously, the lower specificity (67%) of our study may be attributed to challenges in outlining the tumour in ultrasound images for radiomics features analysis. Tumours with poorly defined borders or close to the Cooper’s ligament may be particularly prone to misclassification. However, after removing the patients who had unplanned changes in their chemotherapy, the specificity values increased to 100%.

The strength of the study here includes the prospective design and the relatively complete data of the clinical, imaging, and pathological assessment. As compared to other prediction models using post-treatment scans, refs. [36,37], the current model has the benefit of requiring only the pre-treatment scan. It means that if patients are predicted to be non-responders, clinicians may consider alternate treatment options at the earliest opportunity, though the optimal treatment strategy remains to be defined.

The study here has several limitations. First, the model described here aims to identify patients who will respond poorly to chemotherapy and may benefit from a change in treatment. However, it is not known whether the predicted non-responders have tumours which are resistant to chemotherapy only, or their tumours are so aggressive that they would not respond to any treatment modality. To answer this question, future clinical trials should randomize patients who are predicted to be non-responders to either chemotherapy or other treatment options of physicians’ choices such as hormonal treatment, targeted therapy, radiotherapy, or upfront surgery if feasible. Predictive tools like ours are essential to identify such patients for enrolment to clinical trials which may shed light on the optimal management strategy.

Second, the chemotherapy regimen in both the development and validation cohort was heterogeneous. Our data came from patients who were given a dose-dense regimen given every 2 weeks, a standard regimen given every 3 weeks, sequential anthracycline and taxane-based chemotherapy, and in a minority of patients taxane-based chemotherapy only. Our data also included patients who had HER-2 overexpressed tumours and received trastuzumab. The response to chemotherapy with or without target therapy may depend on the schedule and drugs. It could be argued that a prediction model works best when we included only selected regimen in the development or validation cohort. However, we chose to include patients receiving different chemotherapy regimen to reflect the wide variety of chemotherapy regimens received by patients in the real world. Such prediction models will have wider external validity than those which were highly selective in the treatment regimen received by patients.

Third, it should be noted that the patient composition in the development cohort and validation cohort were somewhat different. Patients in the development cohort were treated with neoadjuvant chemotherapy from 2009 to 2019. This cohort consisted exclusively of patients who had locally advanced breast tumours, which reflected the major indication for neoadjuvant chemotherapy at that time. Following the publication of landmark trials that showed the benefit of adapting the adjuvant treatment based on the response to neoadjuvant treatment, neoadjuvant chemotherapy had become the standard of care in selected patients with early-stage HER-2 overexpressed or triple negative breast cancer [38,39]. Patients in the validation cohort were treated between 2018 and 2021 and included those who had relatively smaller (2–5 cm) tumours which were either HER-2 overexpressed or triple negative. Due to the small sample size, it was not possible to perform subgroup analysis of the predictive accuracy in patients with locally advanced tumours and early-stage tumours (AJCC 7th edition, clinical stage T2N0/1 or T1N1). This can be an inherent limitation of any prediction model of the treatment response in breast cancer, in which the management algorithm is rapidly evolving overtime.

Fourth, some tumours had poorly defined boundary and the delineation of tumour for radiomics features analysis or measurement of its size could be subjective. This would extend to analysis using either CT or MRI where these patients also had ill-defined boundaries. In the study here, the region of interest was defined by a sonographer and verified by a breast radiologist or the principal investigator. We did not collect data regarding the concordance rate of region of interest delineation such as the intraclass correlation coefficient (ICC) and the concordance correlation coefficient (CCC) in the work, however, this has been addressed in previous work [40]. Non-mass enhancing areas in MRI were not studied in the work here. It has also been recognized from ongoing research that the presence of DCIS and likely its micro calcifications result in misclassifications of data.

## 5. Future Research

Moving forward, future research may focus on increasing patient population, stratifying the analysis based on treatment type, and incorporating additional information such as the gene expression profile in model development training process to improve the generalizability, robustness, and accuracy of the response prediction. The management of breast cancer, particularly the adjuvant treatment, has become more personalized over time. Gene expression profile, Ki-67 status, and the presence of specific genetic mutation have all been used to guide the choice of adjuvant treatment [41,42,43]. Combining radiomics and gene expression profile has the potential to further facilitate personalized treatment in neoadjuvant chemotherapy.

After the initial development and validation of these models, patients should be enrolled to randomized controlled trials that assess whether predicted non-responders to chemotherapy can benefit from early initiation of alternate treatment. When interpreting the results of these future studies, clinicians may need to bear in mind that breast cancer is a heterogeneous disease with multiple subtypes and treatment landscape is changing over time. As a result, a prediction model that was developed for some subtypes of breast cancer patients in the past may not necessarily work well for other subtypes of breast cancer treated in the modern era, unless an external validation study like ours has been performed.

## 6. Conclusions

In conclusion, the work here validated a prediction model for responses to neoadjuvant chemotherapy in patients with breast cancers, based on radiomics features extracted using QUS. Future research should aim to improve the accuracy of prediction especially regarding the region of interest delineation and treatment type and evaluate whether the treatment outcome can be improved in predicted non-responders by switching to other treatment options.

## Figures and Tables

**Table 1:** Patient characteristics.

No	Age	ER/PR/ HER-2	Histology	Grade	Pre-NAC Tumour Size (cm)	Pre-NAC T Stage	Pre-NAC N Stage	Treatment
1	47	+--	IDC	III	3	2	1	AC—Paclitaxel (DD), 8 cycles
2	37	++-	IDC	II-III	4.4	3	0	FEC100 x3, then Doce100x3
3	47	++-	IDC	III	6.7	2	1	AC—Paclitaxel (DD), 8 cycles
4	50	+--	IDC	III	1.9	1	0	AC—Paclitaxel (DD), 8 cycles
5	42	+--	IDC	III	8.5	3	1	AC—Paclitaxel (DD), 8 cycles
6	50	++-	IDC	I-II	6.1	2	1	FEC100 x3, then Doce100 + Tras x3
7	35	+++	IDC	II	8	1	1	AC x4, then Paclitaxel (DD) + Tras x4
8	67	--+	IDC	II	4	3	1	FEC100 x3, then Doce100 + Tras x3
9	60	+--	IDC	II	2.4	3	3	FEC100 x3, then Doce100x3
10	45	---	IDC	III	2.7	3	1	AC x4, then Paclitaxel (DD) + Tras x4
11	50	+++	IDC	II	10.7	2	1	AC x4, then Paclitaxel (DD) + Tras x4
12	51	++-	IDC	III	4	2	1	FEC100 x3, then Doce100x3
13	40	+++	IDC	II	5.9	3	1	FEC100 x3, then Doce100x3
14	72	+++	IDC	III	3.5	1	1	FEC100 x3, then Doce100 + Tras x3
15	63	---	IDC	III	4	2	1	AC—Paclitaxel (DD), 8 cycles
16	56	+--	IDC	III	3.5	4	1	Docetaxel-cyclophosphamide, 4 cycles
17	49	+++	IDC	II	4.9	2	0	FEC100 x3, then Doce100 + Tras x3
18	50	++-	IDC	I	3	2	1	AC—Paclitaxel (DD), 8 cycles
19 *	60	---	IDC	II	7.3	2	1	AC—Paclitaxel (DD), 8 cycles
20	41	+--	IDC	II–III	7.5	3	0	AC—Paclitaxel (DD), 8 cycles
21 *	72	---	IMC	III	4.7	2	0	AC—Paclitaxel (DD), 8 cycles
22	53	---	IDC	III	3.1	3	0	AC—Paclitaxel (DD), 8 cycles
23	63	++-	IDC	II	7.4	2	2	FEC100 x3, then Doce100x3
24	64	+++	IDC	I-II	3.4	2	0	FEC100 x3, then Doce100 + Tras x3
25	71	+--	IDC	III	1.7	2	0	Weekly Paclitaxel x12+Tras x4
26	43	---	IDC	II-III	5.6	2	0	AC—Paclitaxel (DD), 8 cycles
27	80	---	IDC	III	10	2	1	AC x4, then weekly paclitaxel x12
28	37	++-	ILC	I-II	12	3	1	FEC100 x3, then Doce100x3
29	27	---	IDC	II-III	1.8	1	0	AC—Paclitaxel, DD, 8 cycles
30	37	+++	IDC	II	2.6	2	0	FEC100 x3, then Doce100x3
31	60	---	IDC	III	3.2	3	1	AC—Paclitaxel (DD), 8 cycles
32	72	++-	IDC	I	3.4	2	0	AC—Paclitaxel (DD), 8 cycles
33	45	++-	IDC	III	9.5	2	1	AC x4, then weekly paclitaxel x12
34	43	---	IDC	III	2.1	2	0	AC—Paclitaxel (DD), 8 cycles
35	53	--+	IDC	III	2.5	2	1	AC x4, then Paclitaxel (DD) + Tras x4
36	54	+-+	IDC	II	3.5	2	0	FEC100 x3, then Doce100 + Tras x3
37	48	+-+	IDC	III	7.5	1	1	FEC100 x3, then Doce100 + Tras x3
38	68	+--	IDC	III	3.6	2	1	AC—Paclitaxel (DD), 8 cycles
39	51	++-	IDC	II	11	3	1	FEC100 x3, then Doce100x3
40	73	++-	IDC	III	7.8	3	2	AC—Paclitaxel (DD), 8 cycles
41	61	--+	IDC	III	2.9	2	0	FEC100 x3, then Doce100 + Tras x3
42	44	+--	IDC	III	6.4	2	0	AC—Paclitaxel (DD), 8 cycles
43	29	++-	IDC	III	8.3	3	1	AC—Paclitaxel (DD), 8 cycles
44	65	---	IDC	II	2.3	2	0	AC—Paclitaxel (DD), 8 cycles
45	43	---	IDC	III	2.4	4	0	AC (DD) x4, then weekly paclitaxel x12
46	32	+++	IDC	II	7.5	3	1	AC (DD) x4, then weekly paclitaxel x12 + Tras x 4
47	68	++-	IDC	II	5	4	1	AC—Paclitaxel (DD), 8 cycles
48	41	++-	IDC	II	2.9	1	1	AC—Paclitaxel (DD), 8 cycles
49	34	---	IDC	III	1.9	1	1	Docetaxel-cyclophosphamide, 4 cycles
50	51	---	IDC	III	1.9	1	1	AC—Paclitaxel (DD), 8 cycles
51	42	--+	IDC	III	3.6	2	1	AC (DD) x4, then weekly paclitaxel x12 + Tras x 4
52	51	++-	IDC	III	3.3	2	0	AC—Paclitaxel (DD), 8 cycles
53 *	31	+++	IDC	III	2.6	2	1	AC (DD) x4, then weekly paclitaxel x12 + Tras x 4
54	44	--+	IDC	III	3.7	2	0	FEC100 x3, then Doce100 + Tras x3
55	47	++-	IDC	III	4.8	2	1	AC—Paclitaxel (DD), 8 cycles
56	34	++-	IDC	II	3.2	2	1	AC—Paclitaxel (DD), 8 cycles

* had a change in neoadjuvant treatment following the disclosure of prediction result. The treatments were given every 3 weeks unless otherwise specified. ER = estrogen receptor; PR = progesterone receptor; HER-2 = human epidermal growth factor 2; NAC = neoadjuvant chemotherapy; IDC = invasive ductal carcinoma; ILC = invasive lobular carcinoma; IMC = invasive mammary carcinoma; AC = doxorubicin–cyclophosphamide; DD = dose dense; FEC 100 = 5-fluouracil, epirubicin 100 mg/m^2^, cyclophosphamide; Doce100 = docetaxel 100 mg/m^2^; Tras = trastuzumab. The status of hormone receptors—estrogen receptor (ER), progesterone receptor (PR), and human epidermal growth factor receptor 2 (HER-2)—is indicated in this table as positive (+) or negative (-).

**Table 2:** Predicted and actual response of all patients.

No	Post-NAC Tumour Size (cm)	Overall Cellularity	RCB Score	Actual Response	Predicted Response
1	2.7	50	2.24	NR	NR
2	2.5	11.25	2.92	R	R
3	3	3	2.51	R	R
4	1.3	75	1.98	R	NR
5	0	0	0	R	R
6	5.28	13.3	3.51	R	R
7	5.7	0.2	0.92	R	R
8	0	15	0	R	R
9	7.2	5	2.72	R	R
10	2.5	3	1.27	R	R
11	2.4	9	3.24	R	R
12	2	5	2.36	R	NR
13	6	0	0	R	R
14	1.2	0	0	R	R
15	0.5	40	1.58	R	R
16	1.9	80	3.09	R	R
17	2.5	10	1.59	R	R
18	3.2	10	3.09	R	NR
19 *	4	10	3.01	NR	R
20	2	0	0	R	R
21 *	3.5	-	-	NR	NR
22	1.5	0	0	R	R
23	7.5	15	3.82	R	R
24	1.7	5	no score	R	R
25	0	0	0	R	R
26	1.8	1	0.37	R	R
27	4	10	3.20	R	R
28	8	15	3.62	R	R
29	1.5	10	1.29	R	R
30	2.5	10	2.35	R	R
31	2.5	30	1.94	R	R
32	1.5	30	2.87	R	R
33	4	25	1.99	R	R
34	0.2	10	0.95	R	R
35	2.5	3.66	0.92	R	R
36	0	0	pCR	R	R
37	0.65	5	2.41	R	R
38	4	40	3.83	R	R
39	8.85	6.5	3.56	R	R
40	4.5	1	1.2	R	R
41	1.4	10	1.33	R	R
42	4	0	pCR	R	R
43	8	2	2.72	R	R
44	0.9	15	1.43	R	R
45	2	0	0	R	R
46	6.6	5.5	3.48	R	R
47	2	12.5	1.60	R	R
48	2.8	25	3.07	R	R
49	1.3	0	pCR	R	R
50	2.5	70	3.88	NR	NR
51	3.5	1	0.78	R	R
52	5.5	0.05	0.72	R	R
53 *	7.86	1.72	1.12	NR	R
54	0	0	pCR	R	R
55	2	75	3.85	R	R
56	6.5	15	3.12	NR	NR

* had a change in neoadjuvant treatment following the disclosure of prediction result. RCB = residual cancer burden; R = responder; NR = non-responder; NAC = neoadjuvant chemotherapy; pCR = pathological complete response.

## Data Availability

Data collected and analyzed in this study are available from the Sunnybrook Research Institute Research Ethics Board approved study “Pilot Investigation of Ultrasound Imaging and Spectroscopy and Ultrasound Imaging of Vascular Blood Flow as Early Indicators of Locally Advanced Breast Cancer Response to Neoadjuvant Treatment”. Since these are patient data, the authors are legally bound to keep them confidential. Data can be made available upon request and review by Institutional Review Board (IRB). Data requests may be sent to Dr. Kullervo Hynynen, vice-president, Research & Innovation, Sunnybrook Research Institute (khynynen@sri.utoronto.ca).

## Supplementary Materials

The following supporting information can be downloaded at: https://www.mdpi.com/article/10.3390/jimaging11040109/s1, Figure S1: The QUS, GLCM based texture and texture-derivative parameter estimation from the ultrasound data; Figure S2: Confusion matrix of treatment response prediction results; Table S1: List of features used in treatment response prediction model development during training process; Table S2: Optimal features selected for tumor response classification using SVB-RBF classifier based on ultrasound QUS-Texture-Derivate and molecular subtype; Table S3: Patient characteristics of the population used to develop treatment response prediction model in 2023.
