# Supplementary material for: Validation of Quantitative Ultrasound and Texture Derivative Analyses-Based Model for Upfront Prediction of Neoadjuvant Chemotherapy Response in Breast Cancer
Source: J Imaging. 2025 Apr 3;11(4):109. doi: 10.3390/jimaging11040109 (PMC12027888; doi:10.3390/jimaging11040109)
Supplement: Supplementary file 1 [file jimaging-11-00109-s001.zip › jimaging-3499312-supplementary.pdf]

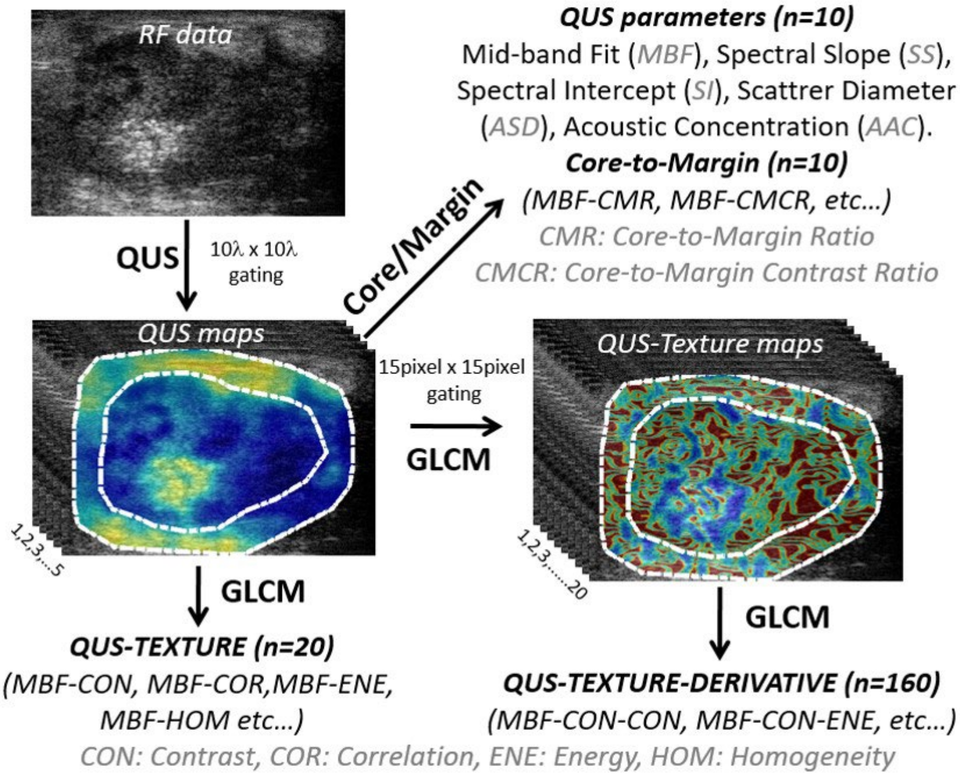

Supplementary Figure S1. The QUS, GLCM based texture and texture-derivative parameter estimation from the ultrasound data are presented. QUS: Quantitative Ultrasound technique; GLCM: grey-level co-occurrence matrix method. QUS maps: MBF, SS, SI, ASD, and AAC parametric maps (5 QUS maps). QUS –Texture maps: MBF-CON, MBF-COR, MBF-EN, MBF-HOM texture maps, etc. (20 QUS-Texture maps). Adapted from Sannachi et al., Supplementary material (Sannachi L, Osapoetra LO, DiCenzo D, *et al.* A priori prediction of breast cancer response to neoadjuvant chemotherapy using quantitative ultrasound, texture derivative and molecular subtype. *Sci Rep* 2023; **13**: 22687.).

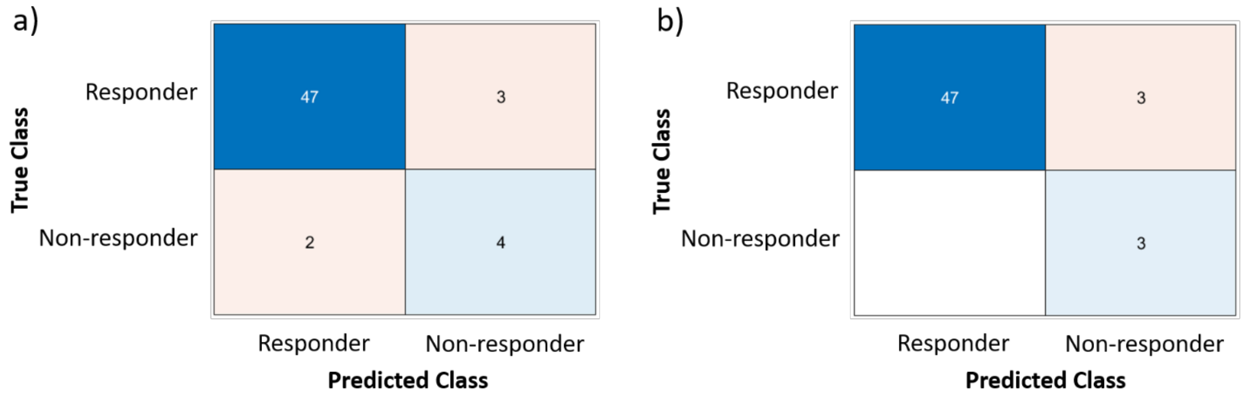

Supplementary Figure S2: *Confusion matrix of treatment response prediction results: a) including the patients with unplanned changes in their chemotherapy treatment, and b) excluding those patients.*

Supplementary Table S1: List of features used in treatment response prediction model development during training process.

No	Feature title	Features
1	Ultrasound propagation property	attenuation
2	Quantitative ultrasound parameters	*Core* MBF
3		*Core* SS
4		*Core* SI
5		*Core* ASD
6		*Core* AAC
7		*Margin* MBF
8		*Margin* SS
9		*Margin* SI
10		*Margin* ASD
11		*Margin* AAC
12	Core-to-Margin parameters	*CMR*-MBF
13		*CMR*-SS
14		*CMR*-SI
15		*CMR*-ASD
16		*CMR*-AAC
17		*CMCR*-MBF
18		*CMCR*-SS
19		*CMCR*-SI
20		*CMCR*-ASD
21		*CMCR*-AAC
22	Texture parameters	*Core* MBF-CON
23		*Core* MBF-COR
24		*Core* MBF-ENE

Supplementary Table S1 (continued)

25		*Core* MBF-HOM
26		*Core* SS-CON
27		*Core* SS-COR
28		*Core* SS-ENE
29		*Core* SS-HOM
30		*Core* SI-CON
31		*Core* SI-COR
32		*Core* SI-ENE
33		*Core* SI-HOM
34		*Core* ASD-CON
35		*Core* ASD-COR
36		*Core* ASD-ENE
37		*Core* ASD-HOM
38		*Core* AAC-CON
39		*Core* AAC-COR
40		*Core* AAC-ENE
41		*Core* AAC-HOM
42	Texture derivative parameters	*Core* MBF-CON-CON
43		*Core* MBF-COR-CON
44		*Core* MBF-ENE-CON
45		*Core* MBF-HOM-CON
46		*Core* SS-CON-CON
47		*Core* SS-COR-CON
48		*Core* SS-ENE-CON
49		*Core* SS-HOM-CON
50		*Core* SI-CON-CON
51		*Core* SI-COR-CON
52		*Core* SI-ENE-CON
53		*Core* SI-HOM-CON

Supplementary Table S1 (continued)

54	Texture derivative parameters	*Core* ASD-CON-CON
55		*Core* ASD-COR-CON
56		*Core* ASD-ENE-CON
57		*Core* ASD-HOM-CON
58		*Core* AAC-CON-CON
59		*Core* AAC-COR-CON
60		*Core* AAC-ENE-CON
61		*Core* AAC-HOM-CON
62		*Core* MBF-CON-COR
63		*Core* MBF-COR-COR
64		*Core* MBF-ENE-COR
65		*Core* MBF-HOM-COR
66		*Core* SS-CON-COR
67		*Core* SS-COR-COR
68		*Core* SS-ENE-COR
69		*Core* SS-HOM-COR
70		*Core* SI-CON-COR
71		*Core* SI-COR-COR
72		*Core* SI-ENE-COR
73		*Core* SI-HOM-COR
74		*Core* ASD-CON-COR
75		*Core* ASD-COR-COR
76		*Core* ASD-ENE-COR
77		*Core* ASD-HOM-COR
78		*Core* AAC-CON-COR
79		*Core* AAC-COR-COR
80		*Core* AAC-ENE-COR
81		*Core* AAC-HOM-COR
82		*Core* MBF-CON-ENE

Supplementary Table S1 (continued)

83	Texture derivative parameters	*Core* MBF-COR-ENE
84		*Core* MBF-ENE-ENE
85		*Core* MBF-HOM-ENE
86		*Core* SS-CON-ENE
87		*Core* SS-COR-ENE
88		*Core* SS-ENE-ENE
89		*Core* SS-HOM-ENE
90		*Core* SI-CON-ENE
91		*Core* SI-COR-ENE
92		*Core* SI-ENE-ENE
93		*Core* SI-HOM-ENE
94		*Core* ASD-CON-ENE
95		*Core* ASD-COR-ENE
96		*Core* ASD-ENE-ENE
97		*Core* ASD-HOM-ENE
98		*Core* AAC-CON-ENE
99		*Core* AAC-COR-ENE
100		*Core* AAC-ENE-ENE
101		*Core* AAC-HOM-ENE
102		*Core* MBF-CON-HOM
103		*Core* MBF-COR-HOM
104		*Core* MBF-ENE-HOM
105		*Core* MBF-HOM-HOM
106		*Core* SS-CON-HOM
107		*Core* SS-COR-HOM
108		*Core* SS-ENE-HOM
109		*Core* SS-HOM-HOM
110		*Core* SI-CON-HOM
111		*Core* SI-COR-HOM

Supplementary Table S1 (continued)

112	Texture derivative parameters	*Core* SI-ENE-HOM
113		*Core* SI-HOM-HOM
114		*Core* ASD-CON-HOM
115		*Core* ASD-COR-HOM
116		*Core* ASD-ENE-HOM
117		*Core* ASD-HOM-HOM
118		*Core* AAC-CON-HOM
119		*Core* AAC-COR-HOM
120		*Core* AAC-ENE-HOM
121		*Core* AAC-HOM-HOM
122		*Margin* MBF-CON-CON
123		*Margin* MBF-COR-CON
124		*Margin* MBF-ENE-CON
125		*Margin* MBF-HOM-CON
126		*Margin* SS-CON-CON
127		*Margin* SS-COR-CON
128		*Margin* SS-ENE-CON
129		*Margin* SS-HOM-CON
130		*Margin* SI-CON-CON
131		*Margin* SI-COR-CON
132		*Margin* SI-ENE-CON
133		*Margin* SI-HOM-CON
134		*Margin* ASD-CON-CON
135		*Margin* ASD-COR-CON
136		*Margin* ASD-ENE-CON
137		*Margin* ASD-HOM-CON
138		*Margin* AAC-CON-CON
139		*Margin* AAC-COR-CON
140		*Margin* AAC-ENE-CON

Supplementary Table S1 (continued)

141	Texture derivative parameters	*Margin* AAC-HOM-CON
142		*Margin* MBF-CON-COR
143		*Margin* MBF-COR-COR
144		*Margin* MBF-ENE-COR
145		*Margin* MBF-HOM-COR
146		*Margin* SS-CON-COR
147		*Margin* SS-COR-COR
148		*Margin* SS-ENE-COR
149		*Margin* SS-HOM-COR
150		*Margin* SI-CON-COR
151		*Margin* SI-COR-COR
152		*Margin* SI-ENE-COR
153		*Margin* SI-HOM-COR
154		*Margin* ASD-CON-COR
155		*Margin* ASD-COR-COR
156		*Margin* ASD-ENE-COR
157		*Margin* ASD-HOM-COR
158		*Margin* AAC-CON-COR
159		*Margin* AAC-COR-COR
160		*Margin* AAC-ENE-COR
161		*Margin* AAC-HOM-COR
162		*Margin* MBF-CON-ENE
163		*Margin* MBF-COR-ENE
164		*Margin* MBF-ENE-ENE
165		*Margin* MBF-HOM-ENE
166		*Margin* SS-CON-ENE
167		*Margin* SS-COR-ENE
168		*Margin* SS-ENE-ENE
169		*Margin* SS-HOM-ENE

Supplementary Table S1 (continued)

170	Texture derivative parameters	*Margin* SI-CON-ENE
171		*Margin* SI-COR-ENE
172		*Margin* SI-ENE-ENE
173		*Margin* SI-HOM-ENE
174		*Margin* ASD-CON-ENE
175		*Margin* ASD-COR-ENE
176		*Margin* ASD-ENE-ENE
177		*Margin* ASD-HOM-ENE
178		*Margin* AAC-CON-ENE
179		*Margin* AAC-COR-ENE
180		*Margin* AAC-ENE-ENE
181		*Margin* AAC-HOM-ENE
182		*Margin* MBF-CON-HOM
183		*Margin* MBF-COR-HOM
184		*Margin* MBF-ENE-HOM
185		*Margin* MBF-HOM-HOM
186		*Margin* SS-CON-HOM
187		*Margin* SS-COR-HOM
188		*Margin* SS-ENE-HOM
189		*Margin* SS-HOM-HOM
190		*Margin* SI-CON-HOM
191		*Margin* SI-COR-HOM
192		*Margin* SI-ENE-HOM
193		*Margin* SI-HOM-HOM
194		*Margin* ASD-CON-HOM
195		*Margin* ASD-COR-HOM
196		*Margin* ASD-ENE-HOM
197		*Margin* ASD-HOM-HOM
198		*Margin* AAC-CON-HOM

Supplementary Table S1 (continued)

199	Texture derivative parameters	*Margin* AAC-COR-HOM
200		*Margin* AAC-ENE-HOM
201		*Margin* AAC-HOM-HOM
202	Tumour Molecular Subtype	ERBB2+ (ER-,PR-,HER2+)
203		Triple Negative (ER-.PR-,HER2-)
204		Luminal-A (ER+ and/or PR+,HER2-)
205		Luminal-B (ER+ and/or PR+, HER2+)

MBF: mid-band fit, SS: spectral slope, SI: spectral intercept, ASD: acoustic scatter diameter, AAC: average acoustic scatterer, CMR: Core-to-margin ratio, CMCR: core-to-margin contrast ratio, CON: contrast, COR: correlation, HOM: homogeneity, ENE: energy, ER: estrogen receptor, PR: progesterone receptor, HER2: human epidermal growth factor receptor 2.

Supplementary Table S2: Optimal features selected for tumor response classification using SVB-RBF classifier based on ultrasound QUS-Texture-Derivate and molecular subtype.

**No.**	Optimal feature set
1	Luminal-A
2	*Core* SI-COR-HOM
3	*Margin* AAC-CON-COR
4	*Core* MBF-CON-COR
5	*Core* SI-COR-ENE
6	*Margin* AAC-COR-CON
7	*Margin* MBF-CON-COR
8	*Core* SS-COR-CON
9	*Core* MBF-ENE-HOM
10	*Margin* AAC-COR-HOM

MBF: mid-band fit, SS: spectral slope, SI: spectral intercept, AAC: average acoustic scatterer, CON: contrast, COR: correlation, HOM: homogeneity, ENE: energy

Supplementary Table S3. Patient characteristics of the population used to develop treatment response prediction model in 2023 [1] (Refer Figure 1 in the main manuscript)

No	Age	Meno pausal status	Pre-Tx tumour size (cm)	His tolo gy	Tumour Grade	ER/ PR/ Her2	Treatment	Post-Tx tumo ur size	Response
1	53	post	5.4	IDC	I	--+	FECD + TRA	0.0	R
2	53	pre	7.3	IDC	I	++-	DE	7.0	R
3	39	pre	5.3	IDC	II	+++	DCB+TRA	2.7	R
4	50	pre	4.6	IDC	III	+++	ACT+TRA	0.0	R
5	31	pre	5.0	IDC	I	++-	ACT	1.4	R
6	46	pre	8.0	IDC	III	---	ACT	6.4	NR
7	39	pre	10.0	IDC	II	+++-	FECD	8.0	R
8	43	post	8.0	IDC	II	+++	ACT+TRA	0.0	R
9	48	pre	4.9	IDC	III	++-	ACT	1.4	R
10	36	pre	5.8	IDC	III	+++	ACT	11.4	NR
11	40	post	4.4	IDC	III	---	ACT	0.0	R
12	62	post	10.0	IDC	III	---	D+XRT	0.0	R
13	36	post	8.0	IDC	III	+++	ACT+TRA	0.0	R
14	59	post	6.0	IDC	III	--+	ACT	2.6	R
15	65	pre	6.7	IDC	II	+--	ACT+TRA	5.5	NR
16	38	pre	9.2	IDC	II	++-	ACT	4.5	R
17	53	pre	11.7	IMC	III	---	ACT	9.4	NR
18	48	pre	9.0	IDC	II	+++	ACT	5.0	R
19	50	pre	5.3	IDC	III	---	ACT	4.0	R
20	49	peri	12.0	IDC	III	--+	D+TRA	0.0	R
21	46	pre	7.0	IDC	III	---	ACT	0.0	R
22	40	post	3.0	IDC	III	-++	ACT+TRA	0.0	R
23	56	pre	3.2	IDC	II	-++	ACT+TRA	0.2	R
24	49	pre	5.6	IDC	II	--+	ACT+TRA	0.1	R

Table S3 continue…

25	47	post	5.2	IDC	II	++-	FECD	6.5	R
26	52	pre	4.1	IDC	N/A	++-	ACT	0.0	R
27	44	pre	9.9	IDC	III	+++	ACT+TRA	2.0	R
28	38	post	9.0	IDC	II	++-	ACT	2.9	R
29	58	pre	1.9	IDC	III	---	ACT	0.0	R
30	36	pre	12.0	IDC	I	++-	ACT	8.0	R
31	38	pre	8.0	IDC	III	--+	ACT	0.0	R
32	47	post	9.9	IDC	II	++-	ACT	18.0	NR
33	57	post	5.5	IDC	III	---	ACT	0.0	R
34	59	peri	4.7	IDC	N/A	++-	ACT	2.1	R
35	47	pre	7.4	IDC	N/A	--+	ACT+TRA	0.0	R
36	55	pre	12.8	IDC	II	++-	ACT	17.0	NR
37	62	post	10.0	IDC	III	+++	FECD	7.0	R
38	32	pre	7.0	IMC	N/A	+++	ACT+TRA	7.4	R
39	38	pre	2.5	IDC	III	---	ACT	3.8	NR
40	45	pre	6.0	IDC	I	+++	ACT+TRA	4.8	NR
41	55	post	10.5	IDC	III	---	ACT	0.1	R
42	59	post	8.0	IDC	II	+-+	FECD+TRA	0.0	R
43	45	pre	3.8	IDC	II	++-	ACT	0.5	R
44	37	pre	3.6	IDC	III	++-	ACTA	2.2	R
45	50	pre	9.0	IDC	II	+++	ACT+TRA	1.2	R
46	54	peri	3.6	IDC	N/A	++-	DC	1.7	R
47	55	pre	1.6	IMC	I	+--	DC	1.2	NR
48	50	post	7.3	IDC	III	---	FECD	2.1	R
49	55	post	3.4	IDC	III	---	ACT	1.8	R
50	32	post	2.7	IDC	II	+-+	ACT+TRA	0.1	R
51	64	post	8.7	ILC	II	++-	FECD	19.0	NR
52	67	post	2.5	IDC	II	---	FECD	3.2	R
53	52	post	2.6	IDC	II	---	FECD	2.5	R

Table S3 continue…

54	56	pre	7.0	IDC	II	+++	ACT+TRA	8.4	NR
55	45	post	2.3	IDC	N/A	+++	FECD+TRA	0.0	R
56	59	post	4.9	IDC	II	++-	FECD	2.8	NR
57	67	pre	7.4	IDC	III	++-	FECD	3.3	R
58	49	pre	2.1	IDC	II	+-+	ACT+TRA	0.0	R
59	62	pre	6.3	IDC	II	---	ACT	12.6	NR
60	58	post	5.2	IDC	I	+++	ACT+TRA	3.4	R
61	58	pre	4.0	IMC	III	--+	DCB+TRA	0.0	R
62	45	pre	4.0	IDC	II	++-	ACT	3.0	NR
63	29	pre	4.2	IDC	III	++-	ACT	4.0	NR
64	79	post	3.9	IDC	II	--+	ACT+TRA	0.1	R
65	42	pre	9.6	IDC	N/A	++-	FECD	3.0	R
66	66	post	3.0	IDC	III	+-+	FECD+TRA	2.4	NR
67	38	pre	5.0	IDC	III	---	ACT	5.0	NR
68	40	pre	11.7	IDC	III	+++	ACT+TRA	1.3	R
69	53	post	8.8	IDC	II	---	FECD	2.5	R
70	47	pre	3.5	IDC	II	++-	ACT	4.0	NR
71	57	peri	3.9	IDC	II	+--	ACT	3.3	NR
72	54	post	5.0	IDC	II	++-	ACT	0.0	R
73	63	post	3.0	IDC	II	++-	ACT	0.5	R
74	31	pre	9.5	IDC	II	++-	ACT	0.6	R
75	47	peri	2.4	IDC	III	---	ACT	0.0	R
76	41	pre	7.9	IDC	II	--+	ACT+TRA	0.2	R
77	43	pre	6.6	IDC	II	++-	ACT	4.0	R
78	38	pre	4.8	IDC	III	---	ACT	0.0	R
79	69	post	4.3	IDC	I	++-	FECD	2.0	R
80	51	post	4.2	IDC	III	--+	ACT+TRA	0.0	R
81	53	post	5.6	IDC	III	--+	ACT+TRA	0.2	R
82	55	post	7.9	IDC	II	++-	ACT	12.6	R

Table S3 continue…

83	51	pre	2.2	IDC	N/A	++-	ACT	0.5	R
84	55	post	3.1	IDC	III	+++	FECD+TRA	0.0	R
85	31	pre	4.0	IDC	N/A	+--	ACT	1.7	R
86	41	pre	2.2	ILC	III	++-	ACT	1.1	R
87	53	Peri	2.3	IDC	II	++-	FECD	0.0	R
88	42	pre	3.1	IDC	N/A	---	ACT	0.0	R
89	31	pre	4.0	IDC	III	---	ACT	0.0	R
90	32	pre	5.6	IDC	III	---	ACT	2.7	R
91	45	pre	8.1	IDC	II	--+	TC+TRA	0.1	R
92	53	pre	5.3	IDC	III	---	FECD	0.0	R
93	58	post	5.3	IDC	III	+++	FECD+TRA	1.5	R
94	38	pre	10.8	IDC	II	++-	FECD	4.9	R
95	72	post	3.3	IDC	II	+--	ACT	0.2	R
96	41	pre	4.5	IDC	III	---	ACT	2.0	R
97	48	pre	5.6	ILC	II	++-	FECD	11.0	NR
98	83	post	7.7	IDC	III	+-+	ACT+TRA	0.0	R
99	51	post	4.5	IDC	III	++-	ACT	3.5	NR
100	43	pre	9.0	IDC	III	---	ACT	3.9	R
101	42	pre	5.0	IMC	III	++-	FECD	8.0	NR
102	60	post	7.2	IDC	III	+++	ACT+TRA	1.0	R
103	42	pre	6.3	IDC	II	++-	FECD	8.4	NR
104	42	pre	4.1	IDC	I	++-	ACT	4.5	NR
105	47	pre	10.4	IDC	II	++-	ACT	4.5	R
106	45	pre	7.3	IDC	II	++-	FECD	1.4	R
107	43	pre	8.5	IMC	II	---	FECD	0.01	R
108	45	pre	6.2	IDC	II	+++	FECD+TRA	4.0	R
109	50	pre	4.2	IDC	II	+++	FECD+TRA	1.9	R
110	62	post	2.1	IDC	I	---	DCB	0.0	R
111	69	post	7.3	ILC	II	+++	ACT+TRA	0.0	R

Table S3 continue…

112	42	pre	6.0	IDC	II	+++	FECD+TRA	2.5	R
113	72	pre	1.3	IDC	II	+++	TC+TRA	0.0	R
114	70	post	5.8	IDC	I	++-	FECD	14.4	NR
115	45	pre	2.0	IDC	I	++-	FECD	0.2	R
116	42	pre	3.8	IDC	III	++-	ACT	1.0	R
117	63	post	8.6	IDC	II	++-	ACT	7.5	NR
118	52	pre	3.1	IDC	II	---	FECD	0.2	R
119	61	post	8.2	IDC	II	++-	ACT	3.0	NR
120	54	post	2.3	IDC	III	---	FECD	0.0	R
121	68	post	2.2	IDC	II	+++	ACT+TRA	0.0	R
122	50	pre	2.5	IDC	III	---	ACT	0.2	R
123	46	pre	2.6	IDC	II	+++	FECD+TRA	0.2	R
124	60	post	6.0	IDC	II	--+	ACT+TRA	0.3	R
125	57	post	3.3	IDC	N/A	--+	FECD+TRA	0.0	R
126	54	post	3.7	IDC	N/A	+-+	ACT	0.0	R
127	67	post	2.5	IDC	III	+++	ACT+TRA	1.4	R
128	55	post	2.7	IDC	III	---	ACT	2.5	R
129	51	post	3.5	IDC	III	+++	ACT+TRA	1.2	R
130	45	post	4.3	IMC	II	++-	FECD	1.9	R
131	45	post	3.2	IMC	III	---	ACT	3.3	NR
132	56	post	2.9	ILC	III	++-	FECD	2.2	NR
133	42	post	2.1	IDC	III	+++	FECD+TRA	0.0	R
134	38	post	1.9	IDC	III	--+	ACT+TRA	0.8	R
135	36	post	2.9	IDC	III	+++	FECD+TRA	0.1	R
136	27	post	11.3	IDC	II	+++	ACT+TRA	2.5	R
137	36	post	5.1	IDC	III	---	ACT	2.5	R
138	66	post	0.8	IDC	I	++-	ACT	0.0	R
139	53	post	3.9	IDC	N/A	++-	ED	3.7	NR
140	62	post	3.5	IDC	I	++-	FECD	0.0	R

Table S3 continue…

141	63	post	3.7	IDC	III	---	ACT	0.0	R
142	81	post	3.8	IDC	III	---	DC	9.0	NR
143	55	post	3.4	IMC	III	---	ACT	1.5	R
144	47	peri	3.9	IDC	III	---	ACT	2.5	R
145	31	post	4.1	IDC	III	++-	ACT	1.2	R
146	34	post	4.1	IDC	III	++-	FECD	3.0	R
147	71	post	3.6	IDC	III	---	ACT	3.0	R
148	46	peri	11.6	ILC	III	+-+	FECD	9.8	R
149	47	post	3.0	IDC	III	+--	ACT	2.7	NR
150	34	post	1.9	IDC	III	---	DC	1.3	R
151	67	post	1.9	IDC	III	---	ACT	0.0	R
152	51	peri	1.9	IDC	III	---	ACT	2.5	NR
153	37	peri	4.4	IDC	III	++-	FECD	2.5	R
154	47	peri	6.7	IDC	III	++-	ACT	3.0	R
155	50	peri	1.9	IDC	III	+--	ACT	1.3	R
156	50	peri	6.1	IDC	II	++-	FECD	5.3	R
157	66	peri	1.9	IDC	N/A	--+	FECD+TRA	0.0	R
158	67	post	4.0	IDC	II	--+	FECD+TRA	1.2	R
159	60	post	2.4	IDC	II	+--	FECD	7.2	R
160	43	peri	5.3	IDC	III	--+	ACT+TRA	0.0	R
161	45	peri	2.7	IDC	III	--+	ACT+TRA	2.5	R
162	49	peri	2.5	IDC	III	+++	ACT+TRA	2.5	NR
163	50	peri	10.7	IDC	II	+++	ACT+TRA	2.4	R
164	51	peri	4.0	IDC	III	++-	FECD	2.0	R
165	40	peri	5.9	IDC	II	++-	FECD	6.0	R
166	72	post	3.5	IDC	III	+++	FECD+TRA	0.0	R
167	51	peri	3.3	IDC	III	++-	ACT	5.5	R
168	63	post	4.0	IDC	III	---	ACT	0.5	R
169	56	post	3.5	IDC	III	+--	DC	1.9	R

Table S3 continue…

170	49	pre	4.9	IDC	II	+++	FECD+TRA	2.5	NR
171	50	pre	3.0	IDC	III	++-	ACT	3.2	NR
172	60	post	7.3	IDC	II	---	ACT	4.0	R
173	58	post	6.3	IDC	III	--+	FECD+TRA	0.9	R
174	53	pre	6.4	IDC	N/A	--+	FECD+TRA	0.3	R
175	41	pre	7.5	IDC	III	+--	ACT	2.0	R
176	72	post	4.7	IDC	III	---	ACT	3.5	NR
177	53	post	3.1	IDC	III	---	ACT	1.5	R
178	63	post	7.4	IDC	II	++-	FECD	7.5	NR
179	64	post	3.4	IDC	II	+++	FECD+TRA	1.7	R
180	71	post	1.7	IDC	III	+-+	ACT+TRA	0.0	R
181	43	post	5.6	IDC	III	---	ACT	1.8	R
182	80	post	2.5	IDC	III	---	ACT	4.0	R
183	37	pre	12.0	IMC	II	++-	FECD	8.0	R
184	27	pre	1.4	IDC	III	---	ACT	1.5	R
185	32	pre	2.6	IDC	II	++-	FECD	4.6	NR
186	55	Post	2.0	IDC	II	+--	ACT	1.0	R
187	60	post	3.2	IDC	III	---	ACT	2.5	NR
188	58	Post	3.2	IDC	III	++	ACT	3.5	NR
189	51	Pre	4.1	IDC	III	++	ACT	1.6	R
190	56	Post	5.2	IMC	III	+-	ACT	0.0	R
191	59	Post	3.1	IDC	III	--+	ACT	0.0	R
192	35	Pre	10.0	IDC	III	++-	ACT	0.0	R
193	74	Post	4.5	IDC	III	---	ACT	1.4	R
194	58	Post	3.0	IDC	III	---	ACT	3.4	NR
195	41	Pre	1.8	IDC	III	+--	ACT	0.0	R
196	57	Post	7.6	IDC	III	---	ACT	5.0	NR
197	46	Peri	6.0	IDC	III	+--	ACT	3.5	R
198	56	Post	4.1	ILC	II	++-	ACT	3.0	NR

Table S3 continue…

199	45	Pre	3.4	IDC	II	-+-	ACT	3.5	NR
200	37	Pre	3.9	IDC	II	++-	ACT	1.3	R
201	52	Post	3.4	IDC	II	++-	ACT	1.5	R
202	66	Post	2.9	IDC	II	++-	ACT	1.0	R
203	57	Post	3.1	ILC	III	++-	ACT	1.9	R
204	44	Pre	1.8	IDC	II	++-	ACT	1.8	NR
205	35	Pre	2.4	IDC	II	+--	ACT	1.0	R
206	49	Pre	6.1	IDC	III	+--	ACT	0.0	R
207	28	Pre	2.7	IDC	III	---	CIS	2.0	NR
208	33	pre	4.5	IDC	N/A	++-	FECD	1.5	R

ILC: invasive lobular carcinoma, IDC: invasive ductal carcinoma, IMC: invasive micropapillary carcinoma, N/A: not available, ACT: Adriamycin and Cytoxan + Taxotere, FECD: Fluorouracil, epirubicin and cyclophosphamide + docetaxel, TC: Taxotere and cyclophosphamide, TRA: trastuzumab, D: docetaxel, XRT: radiation treatment, DC: docetaxel and cyclophosphamide, DCB: docetaxel and carboplatin, ED: epirubicin and docetaxel, CIS: cisplatin, R: Responder, and NR: Non-responder.

References

1. Sannachi, L.; Osapoetra, L.O.; DiCenzo, D.; Halstead, S.; Wright, F.; Look-Hong, N.; Slodkowska, E.; Gandhi, S.; Curpen, B.; Kolios, M.C.; et al. A Priori Prediction of Breast Cancer Response to Neoadjuvant Chemotherapy Using Quantitative Ultrasound, Texture Derivative and Molecular Subtype. *Sci. Rep.* **2023**, doi:10.1038/s41598-023-49478-3.
